# The progression rate of spinocerebellar ataxia type 2 changes with stage of disease

**DOI:** 10.1186/s13023-017-0725-y

**Published:** 2018-01-25

**Authors:** Thais Lampert Monte, Estela da Rosa Reckziegel, Marina Coutinho Augustin, Lucas D. Locks-Coelho, Amanda Senna P. Santos, Gabriel Vasata Furtado, Eduardo Preusser de Mattos, José Luiz Pedroso, Orlando Póvoas Barsottini, Fernando Regla Vargas, Maria-Luiza Saraiva-Pereira, Suzi Alves Camey, Vanessa Bielefeldt Leotti, Laura Bannach Jardim, on behalf of Rede Neurogenética

**Affiliations:** 1 Serviço de Neurologia, Hospital de Clínicas de Porto Alegre, Porto Alegre, Rio Grande do Sul Brazil; 2 Serviço de Genética Médica, Hospital de Clínicas de Porto Alegre, Rua Ramiro Barcelos 2350, Porto Alegre, Rio Grande do Sul 90.035-903 Brazil; 3 Laboratório de Identificação Genética, Hospital de Clínicas de Porto Alegre, Porto Alegre, Rio Grande do Sul Brazil; 4 Grupo de Pesquisa e Pós-Graduação Hospital de Clínicas de Porto Alegre, Porto Alegre, Brazil; 5 Departamento de Estatística, Universidade Federal do Rio Grande do Sul, Porto Alegre, Brazil; 6 Departamento de Medicina Interna, Universidade Federal do Rio Grande do Sul, Porto Alegre, Brazil; 7 Departamento de Bioquímica, Universidade Federal do Rio Grande do Sul, Porto Alegre, Brazil; 8 Programa de Pós-Graduação em Ciências Médicas, Universidade Federal do Rio Grande do Sul, Porto Alegre, Brazil; 9 Programa de Pós-Graduação em Genética e Biologia Molecular, Universidade Federal do Rio Grande do Sul, Porto Alegre, Brazil; 10 Programa de Pós-Graduação em Epidemiologia, Universidade Federal do Rio Grande do Sul, Porto Alegre, Brazil; 11 Faculdade de Medicina, Universidade Federal do Rio Grande do Sul, Porto Alegre, Brazil; 12 Setor de Neurologia Geral e Ataxias. Disciplina de Neurologia Clínica da UNIFESP - Escola Paulista de Medicina, Universidade Federal de São Paulo, São Paulo, Brazil; 13 Laboratório de Epidemiologia de Malformações Congênitas, Fundação Oswaldo Cruz, Rio de Janeiro, Brazil; 14 Departamento de Genética e Biologia Molecular, Universidade Federal do Estado do Rio de Janeiro, Rio de Janeiro, Brazil; 15 Instituto Nacional de Genética Médica Populacional, Rio de Janeiro, Brazil

**Keywords:** Natural history, NESSCA, Progression rate, SARA, SCAFI, Spinocerebellar ataxia type 2

## Abstract

**Background:**

Spinocerebellar ataxia type 2 (SCA2) affects several neurological structures, giving rise to multiple symptoms. However, only the natural history of ataxia is well known, as measured during the study duration. We aimed to describe the progression rate of ataxia, by the Scale for the Assessment and Rating of Ataxia (SARA), as well as the progression rate of the overall neurological picture, by the Neurological Examination Score for Spinocerebellar Ataxias (NESSCA), and not only during the study duration but also in a disease duration model. Comparisons between these models might allow us to explore whether progression is linear during the disease duration in SCA2; and to look for potential modifiers.

**Results:**

Eighty–eight evaluations were prospectively done on 49 symptomatic subjects; on average (SD), study duration and disease duration models covered 13 (2.16) months and 14 (6.66) years of individuals’ life, respectively. SARA progressed 1.75 (CI 95%: 0.92–2.57) versus 0.79 (95% CI 0.45 to 1.14) points/year in the study duration and disease duration models. NESSCA progressed 1.45 (CI 95%: 0.74–2.16) versus 0.41 (95% CI 0.24 to 0.59) points/year in the same models. In order to explain these discrepancies, the progression rates of the study duration model were plotted against disease duration. Then an acceleration was detected after 10 years of disease duration: SARA scores progressed 0.35 before and 2.45 points/year after this deadline (*p* = 0.013). Age at onset, mutation severity, and presence of amyotrophy, parkinsonism, dystonic manifestations and cognitive decline at baseline did not influence the rate of disease progression.

**Conclusions:**

NESSCA and SARA progression rates were not constant during disease duration in SCA2: early phases of disease were associated with slower progressions. Modelling of future clinical trials on SCA2 should take this phenomenon into account, since disease duration might impact on inclusion criteria, sample size, and study duration. Our database is available online and accessible to future studies aimed to compare the present data with other cohorts.

**Electronic supplementary material:**

The online version of this article (10.1186/s13023-017-0725-y) contains supplementary material, which is available to authorized users.

## Background

The spinocerebellar ataxia type 2 (SCA2) is one of the most common polyglutamine (polyQ) disorders. Caused by a dominant expansion of a CAG repeat tract (CAGexp) at *ATXN2*, SCA2 is related to a polyQ with more than 32–33 glutamines in ataxin-2 [1]. Disease usually starts in adulthood and clinical picture is not homogeneous. Main symptoms are related to cerebellar dysfunction, and include ataxic gait, cerebellar dysarthria as well as dysmetria [2]. Severe saccade slowing and peripheral neuropathy are very frequent and affect more than 50% of case series [3]. Besides, several other manifestations might appear, such as pyramidal findings, extrapyramidal syndromes (including dystonic movements and parkinsonism), lower motor neuron findings, cognitive deterioration, and others [4–6]. *ATXN2* expansion explains most but not all variability in age at onset (AO) of symptoms [1], and it was related to presence of some neurological findings such as dystonic movements and parkinsonism [7]. Mean (SD) age at onset was around 30 to 33 (14) years [8, 9] and median survival was 68 [95% CI: 65–70] years, usually after a wheelchair period [10].

Description of disease progression in SCA2 depends on a comprehensive disease-progression model as well as in other SCAs. Several challenges hamper this, such as heterogeneous subphenotypes evolving in time, rarity, and the long duration of disease. Moreover, effects related to genetic or environmental background cannot be discarded. Clinical scales appropriated to the phenotype, description of disease progression in more than one cohort, and anticipating potential drawbacks from data obtained from short duration clinical studies are some of the questions investigators should keep in mind [11].

As stated before, SCA2 symptoms are very heterogeneous. In spite of that, majority of longitudinal studies followed ataxic manifestations only, as measured by Scale for the Assessment and Rating of Ataxia (SARA) [12], SCA Functional-Index (SCAFI) [13], and Composite-Cerebellar-Functional-Score (CCFS) [14]. The natural history (NH) of SARA has been measured a couple of times in SCA2 patients [15–19]. NH of SCAFI and CCFS were described only once for each, with insufficient or non-significant progression rates [18, 20]. An unique study followed up extra-cerebellar findings by using the inventory of non-ataxic symptoms (INAS); however, non satisfactory results were raised [15, 16].

Most longitudinal observations of neurological scales in SCAs used the study entry as the time correspondent to the start of the measurements. First measurements were considered as baseline, abscissa axis was the chronological time since the beginning of study, and the slope of progression was obtained by comparing these data with those obtained at latter observations, usually at fixed intervals [15, 16, 18, 19]. Other studies chose to add age at onset informed by the individual into the model: in these studies, the abscissa axis presented the whole disease duration [21, 22]. If the actual progression rate of the disease is continuous and linear, the slopes obtained by both models should be similar. In contrast, if slopes obtained with these two models are different, this means that progression is not linear and must be further explored.

Our aims were to describe the progression rate of neurological manifestations in a new SCA2 cohort, as measured by the ataxia scales SARA, SCAFI, and CCFS, and by a comprehensive neurologic scale, the Neurological Examination Score for Spinocerebellar Ataxias (NESSCA) [23, 24]; to explore if progression rates are linear during the whole disease duration since onset of gait ataxia; and to look for potential modifiers of disease progression.

## Methods

Symptomatic carriers with a molecular diagnosis of SCA2, under care in outpatient clinics of University hospitals of Porto Alegre, Rio de Janeiro, and São Paulo, Brazil, were invited to participate in this study.

Investigators trained in the scales (TLM, ERR, MA, ASPS) applied NESSCA, SARA, SCAFI, CCFS, and mini-mental state examination (MMSE) in the participants at baseline and in a second visit planned to occur 12 months later. Data was registered in protected files.

Independent variables under study were the following: age, gender, age at onset of gait ataxia (AOga), age at onset of first symptom (AOfs), disease duration since start of gait ataxia (DDga), disease duration since start of first symptom (DDfs), and the number of CAG repeats in both alleles. Molecular studies were performed as previously described [6]. Phenotypic subgroups were built according to presence or absence of amyotrophy, parkinsonism, dystonia, and cognitive losses, as previously described [7]. They were used as additional independent variables. Briefly, amyotrophy was considered present if fasciculations in regions other than face, or muscle tissue loss were found (items 8 and 15 of NESSCA) [22 23]. Parkinsonism was present if at least two out of three manifestations were documented - bradykinesia, rigidity, and resting tremor (items 11 and 12 of NESSCA) [23, 25]. Dystonia was considered present if dystonic movements impaired in some degree the voluntary movements (at least 2 points on item 10 of NESSCA) [23]. Cognitive decline was considered present according to Folstein criteria for MMSE [26].

### Modeling

Linear growth curve models, i.e., mixed models with intercepts and random slopes, were adjusted to model the relationship between outcomes and time. The annual rate of increase was estimated in two different ways:

- Study duration model: A mean change per studied year. Points in time included in this model were the study entry (first observation was the baseline), and 12 and 24 months later (follow up observations).
- Disease duration model: A mean change since the disease onset, according to patient’s report. In this model, at least three time points were of interest: the time of onset of gait ataxia (baseline), the study entry (first observation), and 12 and 24 months later (follow up observations). The progression rate was that estimated to occur during all disease duration.

These different strategies followed the recommendation of Singer and Willett (2003) [27] of investigating alternative temporal specifications. The progression rate obtained during the study duration model was defined as the standard model in the present analysis. If lopes derived from both models were different, the raised hypothesis was that the progression is highly dependent on disease duration, and then a binary variable would be included in the study duration model, according to the apparent effect of disease duration on shifting the progression rate.

A variance component covariance matrix was used for the intercepts and random slopes. Models were fitted in R 3.2.2 software, using *lme4* package. *P*-values were obtained through likelihood ratio tests, using *Anova* function of *car* package. Bootstrap replicates were used to produce confidence intervals for the fitted curves.

### Ethics approval and consent to participate

The study protocol was approved by the institutional ethical standards committees on human experimentation of all contributing centers (registered as 12–0346 at Comissao de Etica em Pesquisa of our institution, and as 07105712.1.0000.5327 at the Brazilian National platform, Plataforma Brasil). All patients gave written informed consent to participate in the study.

### Consent for publication

Not applicable – this report does not contain any individual persons data.

## Results

Forty-nine SCA2 symptomatic carriers (27 men) were included in the baseline analysis, and thirty-eight follow-up evaluations were done. Clinical and molecular characteristics at baseline were already described [7]. Table 1 summarizes demographic data, genetic and neurological findings at baseline (all similar between genders). The original database was anonymized and is also available for readers (Additional file 1).

**Table 1:** - Demographic, molecular and neurologic features of study population at baseline

N subjects (M/F)	49 (27/22)
Age at first examination (years)	46.35 ± 12.26 (24 to 71)^a^
Age at onset of gait ataxia (years)	33.23 ± 12.37 (12 to 59)^a^
Number of CAG repeats at normal *ATXN2*	22.26 ± 0.80 (22 to 27)^a^
Number of CAG repeats at expanded *ATXN2*	40.35 ± 3.21 (34 to 49)^a^
Disease duration at study entry (years)	12.94 ± 6.66 (2 to 27)^a^
NESSCA at baseline	14.37 ± 4.32 (3 to 27)^a^
SARA at baseline	18.42 ± 8.17 (5 to 33)^a^
Main neurological findings at baseline:	
Gait ataxia	49/49
Sensory losses (at least two altered proofs on lower limbs - pin prick/light touch, hot/cold (discrimination) and vibration sensations	19/43^b^
Pyramidal syndrome (at least two of the following: generalized hyperreflexia, Babinski sign, spastic tonus)	5/49
Dysarthria	48/49
Fasciculations and amyotrophy	7/49
Dystonia (dystonic movements that impair in some degree voluntary movements)	8/49
Parkinsonism (at least two of the following: rigidity, bradychinesia, rest tremor)	17/49
With cognitive decline (MMSE ^c^ < = 24 or 18, if schooling was >5 or <= 5 years)	12/49 19.16 ± 6.9 (4 to 24)^a^
Without cognitive decline	37/49 25.9 ± 2.9 (19 to 30)^a^

^a^ mean and standard deviation (range)

^b^ Six subjects were excluded due to the presence of comorbidities such as diabetes melittus

^c^ MMSE: Mini Mental State Evatuation

### Progression rates

Progression rates were obtained for SARA, NESSCA, CCFS and SCAFI according to the mean change per studied year (study duration model) and to mean change since the disease onset (disease duration model).

SARA progressed 1.75 points/year (95% CI: 0.92–2.57) in the study duration model and only 0.79 points/year (95% CI 0.45 to 1.14) in the disease duration model.

NESSCA progressed 1.45 points/year (CI 95%: 0.74–2.16) in the study duration model and only 0.41 points/year (95% CI 0.24 to 0.59) in the disease duration model.

SCAFI progressed just −0.05 points/year (95% -0.09 to −0.01) in the disease duration model. SCAFI did not present a significant progression in the study duration model, while CCFS did not present significant progressions in both models.

The above results documented that there were differences in the progression rates of SARA and NESSCA when both models were applied. The hypothesis was that the progression rate of these scales was not constant during disease duration. Deltas of SARA and NESSCA observed in one year (the study duration model) were then plotted against disease duration in order to determine a cutoff value for the subsequent stratification of the study duration analysis.

**Fig. 1:**
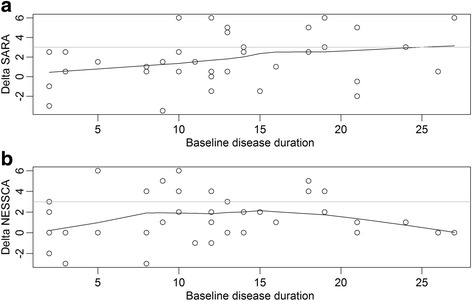
- Differences (deltas) between baseline and follow up observations 12 months later, according to disease duration since onset of gait ataxia. (**a**) Deltas of SARA scores were lower than 3 in the first 10 years of disease duration (**b**) Deltas of NESSCA scores during the first 10 years and after 20 years of disease duration were also lower than those observed in 10–20 years of disease duration

Sixteen out of 38 subjects evaluated in the follow-up had 10 or less years of DD. Figure 1A shows that 2/16 subjects with less than 10 years of disease duration progressed 3 points – and none of them progressed more than that -, whereas 10/22 individuals with more than 10 years of disease duration progressed 3 or more points in SARA scores in one year (chi-square = 4.66, *p* = 0.031). Age, AO and CAGexp at ATXN2 were similar between these DD groups and did not influence their deltaSARAs (data not shown).

**Fig. 2:**
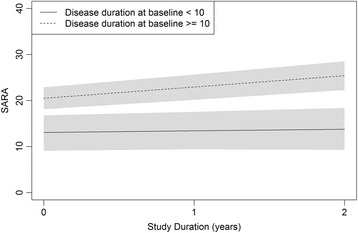
- SARA progression during the study duration, according to disease duration strata

Progression rates of SARA were shown in Fig. 2**,** using the cutoff of 10 years of disease duration to stratify our cohort**.** Symptomatic SCA2 individuals with less and more than 10 years of disease duration progressed 0.35 and 2.45 points/year in SARA scores (*p* = 0.013), respectively.

**Fig. 3:**
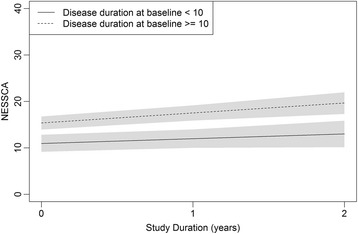
- NESSCA progression during study duration, according to disease duration strata

Figure 1B shows that NESSCA progression is also lower in the first 10 years of disease duration than later on. NESSCA progression turned fast after 10 years, and slowed again after 20 years of disease duration. Due to this finding, and in order to examine the effect of disease stage on the slopes, we have studied further NESSCAs from individuals with less than 20 years of disease duration only, by using the cutoff of 10 years of disease duration. Progression rates of NESSCA were shown in Fig. 3**.** Symptomatic SCA2 individuals with less and more than 10 years of disease duration progressed 1.03 and 2.14 points/year in NESSCA scores (*p* = 0.191), respectively.

### Modifier factors

**Fig. 4:**
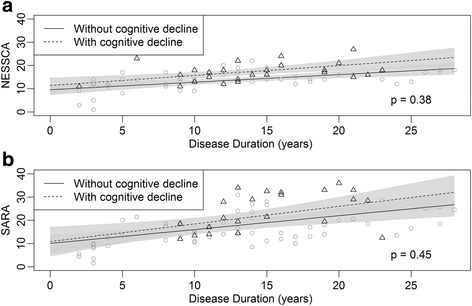
- Disease progression as measured by clinical scales in SCA2 individuals according to disease duration model. (**a**) NESSCA progression. (**b**) SARA progression. Hatched lines describe progression rates of subjects with cognitive decline, while continuous lines describe progression rates of subjects without cognitive decline

Gender, AOga, AOfs, CAGexp at *ATXN2*, and presence/absence of amyotrophy, parkinsonism, dystonic manifestations and cognitive decline at baseline, were studied as potential modifier factor of disease progression according to both models (study duration model and disease duration model). None of them produced significant differences in the progression rates - even using the disease duration strata revealed in Figs. 1 ,2, and 3. Figure 4 shows an example of the NESSCA and SARA progression rates obtained in subjects with and without cognitive decline.

## Discussion

Our results showed that progression rates of SARA and NESSCA were not constant during the long disease duration of SCA2 symptomatic patients. At early phases, i.e., in the first 10 years of the disease, progression rates of both scales were slower than in the following years. This phenomenon might be due to the psychometric characteristics of scales or to biological causes. Whatever the reason, the direct use of linear models during prospective longitudinal observations without paying attention to differences in disease duration might keep these non-linear progressions hidden.

At least nine studies followed SCA2 patients with longitudinal observations [15–19, 28–31]. In most cases where SARA progression was measured, annual worsening was around 1.5 to 1.9 points [15, 16, 18, 19, 30]. The methodology of analysis of two of these former cohorts were similar to that from our group therefore, our results can be compared to those studies [15, 16, 18]. Our observations related to the study duration analysis raised a SARA progression of 1.75 points/year, which is comparable to theirs. However, neither observations related to disease duration (Fig. 1) nor discrepancy of results obtained by the two models has been reported before.

Disease progression of a cohort of 35 SCA2 patients living in France was analysed using mixed models with a random effect for patients and the fixed effects group and time between inclusion and clinical examination [18]. Authors found that SARA worsened 1.3 (0.2) points per year. Factors associated with faster SARA progression were male gender, and patients who were younger at onset. Disease duration and CAGexp did not change SARA progression in that cohort. The Eurosca study included 163 SCA2 patients from several European countries in a longitudinal cohort, and linearity of the progression rate was tested via nested models (likelihood ratio test), followed by an analysis of covariance where the effect of gender, age at onset, disease duration, and repeat length of the expanded allele were tested [15, 16]. SARA worsened 1.40 points per year. Earlier age at onset and longer expanded alleles were associated with faster SARA progression: in the multivariate analysis, age at onset was the only independent factor. Thirty Cuban symptomatic subjects were evaluated four times (baseline, and at 1, 2, and 5 years), in a study that longitudinally followed variations in SARA and in saccades: the exponential progression rate of the SARA score was associated to the CAGexp [19].

Therefore, previous SCA2 cohorts showed effects of gender, early ages at onset or of CAGexp on speeding SARA progression. None detected a difference related to disease duration. In contrast, our cohort showed a trend to associate a faster NESSCA (not SARA) progression to larger CAGexp. These discrepancies can be due to differences in sample sizes - the number of observations impacting on the choice of statistical modeling -, or to truly differences between cohorts with diverse populational origins.

Contrary to previous cohorts, our longitudinal observation was able to pick up a clear effect of disease duration on the slope of progression of SARA and NESSCA. This effect was detected because of the discrepancy between the slopes obtained with the two models: the study duration and the disease duration models. Discrepancy led us to look for deltas distributions (Fig. 1) and a cutoff value was chosen with the empirical data. Both Jacobi et al. [15] and Tezenas du Montcel et al. [18] analysed the data by the study duration, using the time between inclusion and clinical examination as one of the fixed effects. We questioned whether the treatment of disease duration in their model was unable to reveal this variable as a modifier. It is relevant to state that disease duration entered their model as a factor whose interaction with progression rate was tested with a mathematical treatment - either as a continuous or a dichotomous variable, splitted by the median. This procedure fitted totally with the generalized linear mixed model; but it might be insufficient to clarify the problem. A good way to shed light into this problem will be to perform either multicentric studies or a meta-analysis. Our database is available online with the present communication in order to help any of these approaches.

Non linear are as plausible as linear progressions for neurodegenerative diseases and were already clearly proposed for SCA2 [19]. In Huntington disease (HD), another polyQ disorder, progression rates of chorea and of caudate atrophy are not linear. Slopes for caudate atrophy changes with the clinical stage [32]. The annual rate of increase in chorea is greater among individuals with earlier-stage HD than in those with advanced HD [33]. Reasons for non-linearity might include scale limitations and truly natural phenomena. For instance, NESSCA progression seemed to be slower either in the first as well as in the last years of the disease (Fig. 1B). We postulate that the slowdown seen after 20 years of the disease more probably reflects the inability of this scale to measure progression after a certain disease stage. In any case, statistical modeling is an issue for discontinuous deteriorations. In another study, we used markov chains to describe the progression of several neurological findings in SCA3/Machado Joseph disease (SCA3/MJD). Although markov chains are quite uneasy and unfamiliar for clinical researchers, this model disclosed that isolated findings, such as gait ataxia, limb ataxia, dystonic manifestations and others, followed a curvilinear trajectory as the disease progressed [21]. Perhaps the present approach, where the use of mixed models was done in two stracta, splitted by a cutoff for dichotomous (dummy) observations chosen by an immediate, empirical data judged by eye inspection, can be more helpful.

## Conclusions

The present study suggested that the speed of progression of scales SARA and NESSCA is not uniform during the disease process in SCA2, varying according to stage of disease. General progression rates of SARA and NESSCA were either similar to others studies in SCA2 (1.7 points per year in the case of SARA) or very like other SCA (1.45 points per year in the case of NESSCA, similar to the progression found in SCA3/MJD), while general progression of SCAFI and CCFS were non significant, at least in the study duration model. Early phases of disease were associated with slower SARA and NESSCA progressions, when compared to phases after 10 years of disease onset. Future clinical trials on SCA2 should take this into account when estimating sample size/study duration. Moreover, we recommend that disease duration should be included in recruitment criteria. Finally, our database is available online and accessible to future studies aimed to compare our cohort with other databases. A meta-analysis would be the best way to elucidate all events that influence the progression of this disease.

## Additional file

Additional file 1: Database on the Brazilian SCA2 cohort, by Monte et al 2018, Orphanet Journal of Rare Diseases. (XLSX 224 kb)

## Abbreviations

AO: age at onset
AOfs: age at onset of first symptom
AOga: age at onset of gait ataxia
CAGexp: expanded CAG repeat tract
CCFS: Composite-Cerebellar-Functional-Score
DDfs: disease duration since the start of first symptom
DDga: disease duration since the start of gait ataxia
HD: Huntington disease
INAS: inventory of non-ataxic symptoms
MMSE: mini-mental state examination
NESSCA: Neurological Examination Score for Spinocerebellar Ataxias
NH: natural history
polyQ: polyglutamine
SARA: Scale for the Assessment and Rating of Ataxia
SCA: Spinocerebellar ataxia
SCA2: Spinocerebellar ataxia type 2
SCA3/MJD: Spinocerebellar ataxia type 3/Machado-Joseph disease
SCAFI: SCA Functional-Index
SD: standard deviation
